# Postoperative Intussusception in a Neonate with Congenital Cutis Laxa and Huge Hiatal Hernia

**Published:** 2014-04-01

**Authors:** Mohajerzadeh Leily, Sadeghian Naser, Mirshemirani Aliraza, Khaleghnejad Tabari Ahmad, Rouzrokh Mohsen, Jafari Nahid

**Affiliations:** Pediatric Surgery Research Center, Shahid Beheshti University of Medical Sciences, Tehran, Iran

**Keywords:** Congenital cutis laxa, Hiatal hernia, Postoperative intussusceptions

## Abstract

Congenital cutis laxa is a genetically heterogeneous condition presenting in the newborn with loose, redundant skin folds, decreased elasticity of the skin, and general connective tissue involvement. A 2-day-old full term neonate with congenital cutis laxa presented with respiratory distress. Investigations revealed huge hiatal hernia. Patient underwent loose Nissen’s fundoplication. In postoperative period patient developed intussusception which was manually reduced at re-surgery.

## INTRODUCTION

Cutis laxa is an uncommon disorder characterized by degenerative changes in the elastic fibers; it can be inherited (autosomal recessive, autosomal dominant and X-linked recessive) or acquired.[1] Postoperative intussusception is reported in various kinds of operations especially involving retroperitoneal dissection.[2] We herein report a neonate of cutis laxa with hiatus hernia who developed postoperative intussusception.

## CASE REPORT

A-2-day-old full term and normally delivered male neonate with features of congenital cutis laxa was referred with severe respiratory distress (Fig. 1). His facial features included; a broad flat nose with everted nostrils, a short columella, a long upper lip, and everted lower eyelids with large ears. His skin was lax and had a hoarse cry. Hip joint was lax. Physical examination revealed slight tachypnea. Arterial blood gas and electrolytes were normal. Thoraco-abdominal x-ray demonstrated coiled nasogastric tube (NGT) above diaphragm (Fig. 2). Barium swallow showed stomach in the thoracic cavity, and echo was normal. He underwent surgery at 6th day of life. Operative findings revealed an intact diaphragm with a huge combined sliding-rolling (mixed) hernia, containing an upward dislocation of both the cardia and the gastric fundus into thoracic cavity. The gap of the esophageal hiatus was 3 cm. Hiatal hernia was repaired along with loose Nissen’s fundoplication. Immediate postoperative period was normal however he developed abdominal distension on 3rd postoperative day. Abdominal ultrasound revealed only bowel distention. Contrast study with oral gastrographin showed target singe. He was re-explored through pervious incision. An ileoileal intussusception found which was manually reduced (Fig. 3). Patient is doing fine on follow-up.

**Figure:**
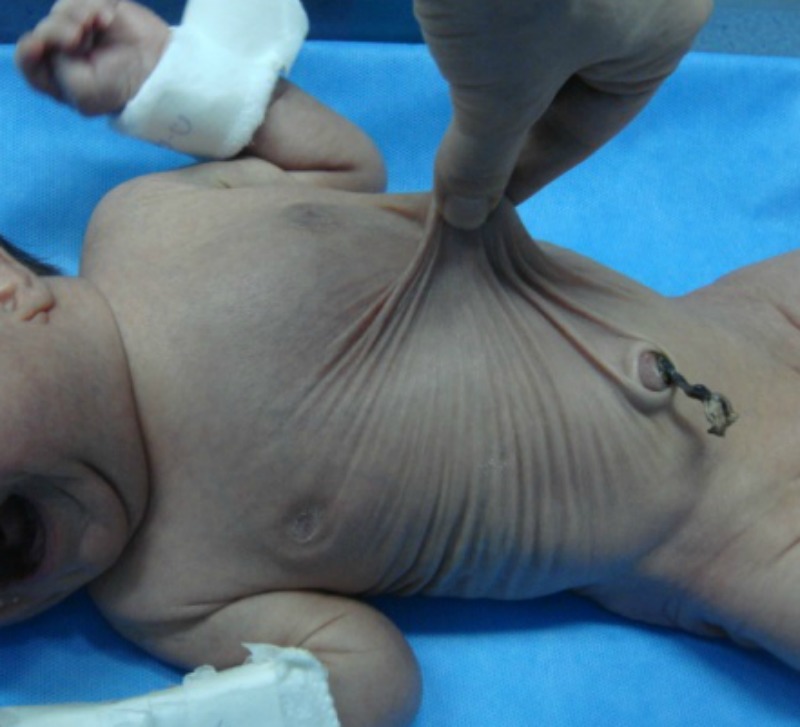
Figure 1:Neonate operated for hiatal hernia with cutis laxa.

**Figure:**
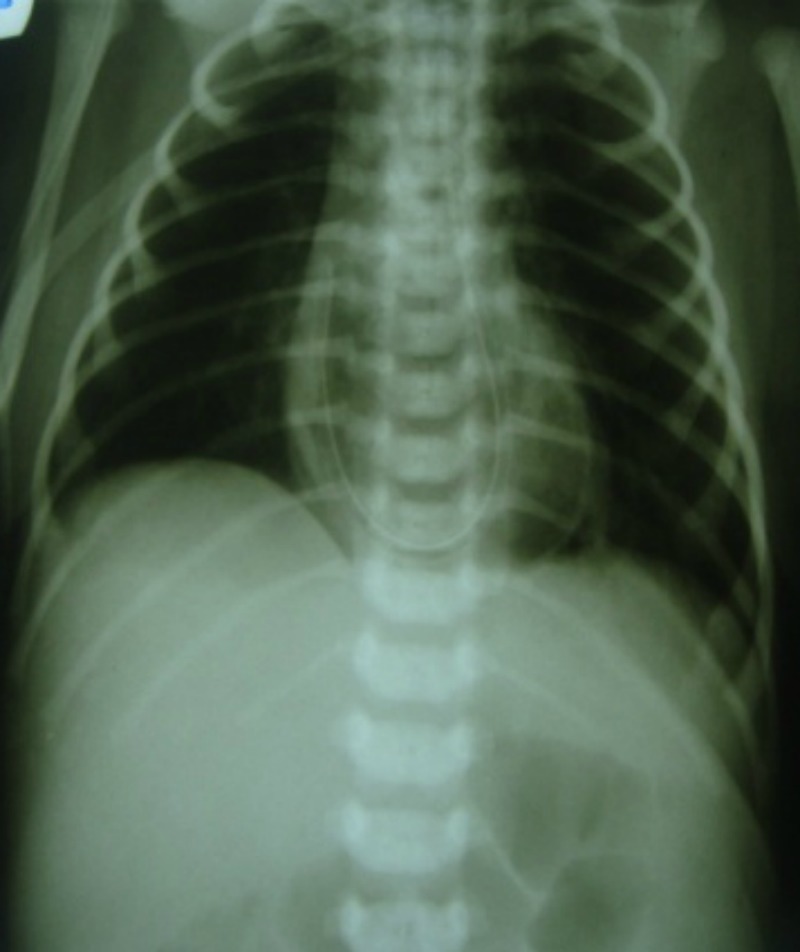
Figure 2:Presence of coiled nasogastric tube above diaphragm.

**Figure:**
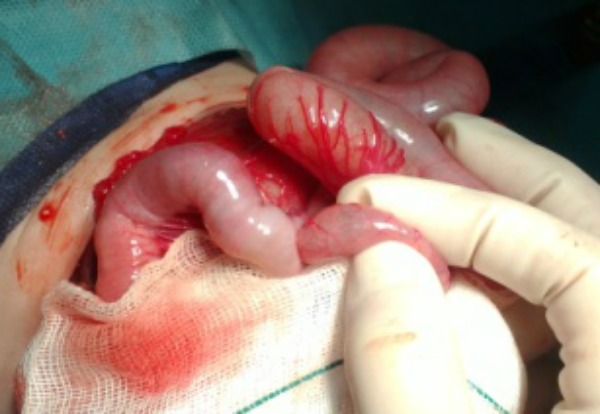
Figure 3:Ileo-ileal intussusception.

## DISCUSSION

Cutis laxa is an acquired or inherited skin disease characterized by wrinkled, inelastic skin. Congenital problems of elastin synthesis and structural defects of extracellular matrix proteins have been described leading to the decreased elasticity and redundant sagging skin.[3] The autosomal recessive cutis laxa is highly heterogeneous with organ involvement as, lung atelectasis and emphysema, diverticulae of the gastrointestinal and genitourinary organs and vascular abnormalities (arterial aneurysms, fibromuscular artery dysplasia and stenosis). Diminution of elastic fibers throughout the dermis and abnormal elastin components by electron microscopy are pathognomonic. [4, 5] Autosomal dominant cutis laxa are often recognized in beginning of childhood with a mild form of cutis laxa with systemic involvement.[1] Vascular abnormalities include arterial aneurysms leading to severe heart failure.[6]. Our case was a congenital cutis laxa that demonstrated vomiting, tachypnea and tachycardia at the birth. He had no family history of this disease.

Kothari et al reported the first infant with cutis laxa coupled with multiple hernias and cardiac disorder.[7] Patient had para-esophageal hernia, right gluteal hernia and ventricular septal defect. He was operated in infancy period .Our case presented on first day of life with respiratory distress and underwent surgery in neonatal period.

Postoperative intussusception may develop in a number of abdominal and extra-abdominal surgeries. It has special predilection for surgeries with retroperitoneal dissection.[2] It is mostly ileo-ileal as in the index case.

## Footnotes

**Source of Support:** Nil

**Conflict of Interest:** None declared

